# Redox Regulation by Protein S-Glutathionylation: From Molecular Mechanisms to Implications in Health and Disease

**DOI:** 10.3390/ijms21218113

**Published:** 2020-10-30

**Authors:** Aysenur Musaogullari, Yuh-Cherng Chai

**Affiliations:** Department of Chemistry, John Carroll University, University Heights, OH 44118, USA; amusaogullari21@jcu.edu

**Keywords:** S-glutathionylation, redox modification, protein post-translational modification, GSH/GSSG, oxidative stress

## Abstract

S-glutathionylation, the post-translational modification forming mixed disulfides between protein reactive thiols and glutathione, regulates redox-based signaling events in the cell and serves as a protective mechanism against oxidative damage. S-glutathionylation alters protein function, interactions, and localization across physiological processes, and its aberrant function is implicated in various human diseases. In this review, we discuss the current understanding of the molecular mechanisms of S-glutathionylation and describe the changing levels of expression of S-glutathionylation in the context of aging, cancer, cardiovascular, and liver diseases.

## 1. Introduction

Reactive oxygen species (ROS) are derivatives of molecular oxygen produced in a wide range of cellular processes [1]. Mitochondrial respiration, for example, is a significant source that generates ROS through the leakage of electrons from donor centers of the electron transport chain to oxygen. Various enzymes, such as NADPH oxidases (NOXs), also generate ROS as part of their normal enzymatic function [2]. The disruption in the homeostatic balance that exists between the production and elimination of ROS results in a phenomenon known as oxidative stress. The elevated levels of ROS during oxidative stress may lead to irreversible oxidation of macromolecules (proteins, lipid, and DNA), and eventual cell and tissue damage. To prevent these harmful effects, both enzymatic and non-enzymatic antioxidant defense mechanisms exist to maintain a steady-state control over ROS production and detoxification. There is growing evidence that links the alteration of the redox metabolism to the progression of diseases [3].

Although historically viewed as harmful byproducts of metabolism, ROS may also function as important second messengers within intracellular redox signaling pathways [4,5]. The major mechanism by which ROS mediate redox regulation is through the modification of target proteins. Specifically, protein sulfhydryl groups (i.e., thiols) have gained particular attention for their ability to transform oxidant signals into biological responses [6,7]. Redox reactions of protein reactive thiols may lead to various post-translational, reversible, and regulatory modifications, including S-sulfenylation, S-nitrosylation, and S-glutathionylation. Moreover, thiols may further oxidize into largely irreversible species, such as sulfinic and sulfonic acids, permanently altering the function of proteins [8]. Chief among these modifications concerning demonstrated physiological significance and prevalence is S-glutathionylation: the reversible formation of mixed disulfides between protein-reactive thiols and the most abundant, non-protein antioxidant glutathione.

In mammalian cells, S-glutathionylation can function as a regulatory mechanism or protect proteins against irreversible oxidation. By introducing the tripeptide glutathione with additional ionic charges into a protein, S-glutathionylation resembles the well-characterized mechanism of protein phosphorylation. The dynamic and reversible nature of S-glutathionylation highlights its ability to function as a redox “switch”, regulating protein function, interactions, and localization [9]. In this review, we focus on the chemical background of thiol modifications and glutathione in redox regulation. We discuss the current understanding of the molecular mechanisms of S-glutathionylation and describe the different levels of expression in S-glutathionylation of specific proteins in the context of aging, cancer, cardiovascular and liver diseases.

## 2. The Biochemistry of Protein Cysteine Residues: A Basic Overview

Cysteine is one of the least abundant, but perhaps the most functionally diverse, of all twenty standard amino acids [10]. Despite its low abundance, cysteine is present within functional sites in proteins where it serves catalytic, regulatory, structural, and other critical functions [11]. In addition, cysteine residues are frequently found in metal-binding sites of proteins forming complexes with metal ions that act as cofactors [12]. These distinct features of cysteine are attributed to the chemical and physical properties of its sulfur-based functional group. The thiol group allows cysteine to participate in unique redox reactions, forming post-translationally modified structures that exhibit significantly altered functions [13].

The human genome encodes about 214,000 cysteines [14]. Cysteine residues can be distributed on protein surfaces exposed to an aqueous environment or deeply buried inside globular domains [15]. In aqueous solution, the thiol side chain of cysteine is subject to deprotonation to form negatively charged thiolate. Although both forms of cysteine have lone pairs of electrons and are thus nucleophilic, thiolates are much more reactive than thiol groups. At any given pH, the ratio of thiol: thiolate is determined by the ionization constant (pK_a_) of the thiol group. The pK_a_ of the unperturbed cysteine thiol is approximately 8.5 [10], indicating that only a relatively small proportion of lone cysteine residues would be in their thiolate forms at physiological pH. However, protein thiols can be strongly influenced by the local microenvironment and exhibit a wide range of pK_a_ values [11]. Additionally, it is essential to note that although a lower pK_a_ means higher availability of thiolates at neutral pH, the nucleophilicity of thiolates is either decreased or unaffected at lower pK_a_ depending on the substrate tested [16].

Under oxidative stress conditions, the one-electron oxidation of a cysteine thiol leads to the generation of the thiyl radical (RS^•^). Free radical species that oxidize thiols to thiyl radicals in vivo include hydroxyl (^•^OH), carbonate (CO_3_^•–^), nitrogen dioxide (^•^NO_2_), superoxide (O_2_^•–^), and peroxyl and phenoxyl radicals [17]. The thiyl radical participates in the catalytic mechanism of several enzymes, including ribonucleotide reductase, which performs the abstraction of the 3′-hydrogen of a nucleotide to convert it to its deoxy-form [18].

The two-electron oxidation of a cysteine thiol with hydrogen or alkyl hydroperoxides yields sulfenic acid intermediates (RSOH) [19]. This mechanism is mediated by an S_N_2 reaction where the cysteine thiolate acts as the nucleophile. Sulfenic acids are highly reactive species that exhibit both nucleophilic and electrophilic character. The condensation of two sulfenic acids to generate a thiosulfinate exemplifies this dual nature [20]. Enzymes that involve a sulfenic acid intermediate in their catalytic mechanism include peroxiredoxin and glutathione peroxidase, both members of the thioredoxin superfamily that catalyze the reduction of hydroperoxides [21].

**Figure 1:**
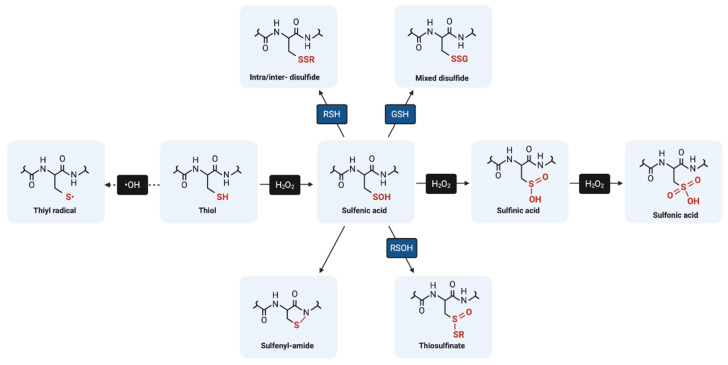
Biologically relevant oxidative post-translational modifications of cysteine.

Sulfenic acids are transient species that can be subject to several alternative fates. Depending on the microenvironment, a sulfenic acid may react with an adjacent thiol to form a disulfide (RSSR’) or it may react with a backbone amide to form a sulfenyl-amide (RSNHR’) [20]. In the absence of these stabilizing groups and under high oxidant concentrations, sulfenic acids are subject to further oxidation to sulfinic acid (RSO_2_H) and sulfonic acid (RSO_3_H), which are more stable, irreversible type modifications generally associated with protein misfolding, degradation, and pathology [22]. The only known exception for the irreversibility of sulfinic acid formation is the ATP-dependent reaction catalyzed by sulfiredoxin, a specialized repair protein that reduces the sulfinic form of peroxiredoxins (cysteine-based peroxidases) back to their sulfenic form [23]. Figure 1 depicts a summary of the biologically relevant oxidative post-translational modifications of cysteine.

It is well established that only particular cysteine residues in proteins are susceptible to oxidative modifications [24,25]. About 10–20% of the total cellular cysteine thiols are readily oxidized under aerobic conditions, while the remaining are thought to be relatively inert to redox reactions [26]. As discussed above, one chemical property that affects the thiol oxidation susceptibility is low pK_a_ [27]. Many biological oxidants, including hydrogen peroxide (H_2_O_2_), react exclusively with the thiolate form of cysteine [28]. Winterbourn et al. show that the reaction rates of low molecular weight thiols with H_2_O_2_ at pH 7.4 increase with decreasing thiol pK_a_ [29]. However, low pK_a_ of a thiol is not the sole determinant of its oxidation susceptibility. This fact is highlighted by comparing protein tyrosine phosphatase 1B (PTP1B) (pKa = 5.4) and peroxiredoxin (pKa = 5.6). Although the pKa of PTP1B and peroxiredoxin are similar, there is a 10^7^-fold difference in the reaction rate constants of H_2_O_2_ with their active sites [29].

Chemical features of the local microenvironment may also affect the reactivity of cysteine. For instance, vicinal polar or positively charged amino acids stabilize the thiolate anion, thereby lowering its pK_a_ and increasing the reactivity of cysteine [30]. Likewise, due to the dipole character of α-helices, cysteine residues located towards near the N-terminal are known to have lower pK_a_ and higher reactivity than cysteines near the C-terminal [8]. Other features that may affect the susceptibility of cysteine residues to undergo oxidation may be related to the accessibility of the solvent, or the type of oxidant reacting with the cysteine thiolate. At present, the molecular basis of the specificity of these reactions remains incompletely understood.

## 3. Low-Molecular Weight Non-Protein Thiols in Redox Regulation: Focus on Glutathione

In the reducing environment of the cytoplasm, low-molecular-weight (LMW) thiols are produced in high concentrations to serve as cofactors, reduce existing oxidative modifications, and form covalent linkages with protein thiols [31,32,33]. One common feature of LMW thiols is that biosynthetically, their thiol functional groups originate from cysteine [34,35]. Besides, almost all organisms contain NADPH-dependent reductases that catalyze the reduction of LMW thiols once they are oxidized [33]. Different organisms utilize different types of LMW thiols. Although cysteine and coenzyme A are present in all organisms, mycothiol and bacillithiol are observed only among Gram-positive bacteria [36,37]. Among eukaryotes, Gram-negative bacteria, and some Gram-positive bacteria, glutathione is the predominant LMW thiol [38].

Glutathione is a ubiquitously distributed tripeptide consisting of three amino acids: glycine, cysteine, and glutamic acid. The thiol group of cysteine is the key functional component of the molecule responsible for its biological activity. The structure of glutathione is unique such that the N-terminal glutamate and cysteine residues are bonded by the γ-carboxyl group of glutamate rather than the conventional peptide bond formation in proteins utilizing the α-carboxyl group [39]. Consequently, this bond’s presence renders glutathione relatively stable and resistant to intracellular degradation in the cell. Initially, it was thought that this unusual bond could only be hydrolyzed by γ-glutamyl transpeptidase (GGT), an essential enzyme localized on the plasma membrane of specific cell types. However, studies have shown that CHAC1 protein found among higher eukaryotes has γ-glutamyl cyclotransferase activity that also specifically degrades intracellular glutathione [40,41,42].

In most cells, glutathione is present at concentrations of 1–10 mM in the cytosol, and at lower concentrations in subcellular organelles, including the nucleus, mitochondria, and endoplasmic reticulum [43]. The tripeptide mainly exists in reduced (GSH) and oxidized forms (glutathione disulfide, GSSG), and the ratio between these (GSH:GSSG) is used as a marker to determine the presence of oxidative stress. Moreover, the GSH:GSSG ratio is crucial to create cellular compartments with distinct redox signatures. For example, under the actively reducing environment of the cytoplasm, with a GSH:GSSG ratio exceeding 100:1, it is difficult to form intra- and intermolecular protein disulfides. However, the endoplasmic reticulum, which has a GSH:GSSG ratio ranging from ~1:1 to 3:1, provides a more oxidative environment to permit oxidative protein folding [44]. Therefore, an optimal GSH/GSSG ratio must be maintained for physiological processes to occur.

Glutathione serves vital functions inside the cell, including but not limited to antioxidant defense, maintenance of redox potential, redox signaling, and regulation of cell growth and death. The pK_a_ of the thiol group of GSH, shown to be around 9.6, confers low reactivity to the molecule [45]. Thus, although glutathione may scavenge ROS and xenobiotic substances directly, an enzyme-catalyzed elimination is faster [43]. Glutathione S-transferases (GSTs) lower the pK_a_ of the thiol group of GSH to catalyze the nucleophilic addition of the tripeptide to xenobiotics [46]. Similarly, most of the glutathione in antioxidant defense is utilized by glutathione peroxidases [47]. These enzymes catalyze the reduction of H_2_O_2_ to H_2_O by the coupled oxidation of GSH to GSSG. Subsequently, GSSG is reduced back to GSH by the action of glutathione reductase and NADPH. Other enzymes such as peroxiredoxins and glutaredoxin that require thiol-reducing equivalents also utilize glutathione as a reductant to restore enzymatic function by keeping their redox-sensitive active sites in the reduced state [48].

## 4. Molecular Mechanisms of Protein S-Glutathionylation

**Figure 2:**
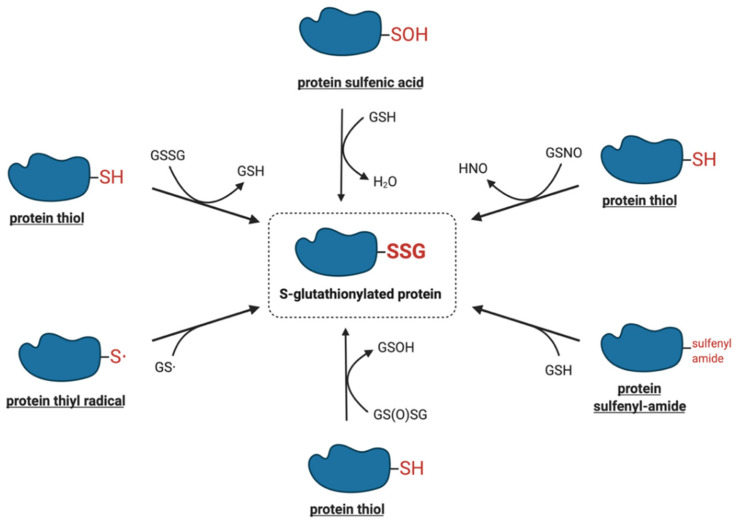
Proposed molecular mechanisms of S-glutathionylation.

The modification of protein-reactive thiols with glutathione was initially thought to be a non-specific reaction induced in pathological, highly oxidative situations or upon exposure to strong oxidants, particularly under in vitro conditions [22]. Recent studies, however, have demonstrated that protein S-glutathionylation is a dynamic process that may also occur under physiological conditions. Here, we discuss a number of chemical mechanisms by which protein thiols can interact with the cellular glutathione pool, leading to their S-glutathionylation. Figure 2 shows the summary of the potential mechanisms of S-glutathionylation that are discussed in more detail below.

### 4.1. Thiol–Disulfide Exchange Mechanism

One of the most intensively studied mechanisms for protein S-glutathionylation involves a thiol-disulfide exchange reaction [49]. The thiol-disulfide exchange mechanism primarily relies on the redox state of cellular glutathione. In principle, oxidative stress could result in the oxidation of GSH molecules to form GSSG, shifting the GSH:GSSG redox balance toward a more oxidizing state. As a result, it was hypothesized that the accumulation of GSSG would lead to a spontaneous thiol–disulfide exchange between protein cysteine thiols and GSSG, thereby yielding the corresponding protein-mixed disulfide along with GSH (Equation (1)). In this mechanism, the extent of protein S-glutathionylation ([PSSG]:[PSH]) is dependent on the intracellular GSH:GSSG ratio. Therefore, the equilibrium constant of the reaction (K_mix_, Equation (2)) represents the specific oxidation potential for the formation of the mixed disulfide.

PSH + GSSG ⇌ PSSG + GSH(1)

(2) $$K_{\mathrm{mix}}=\frac{\left[\mathrm{PSSG}\right]\left[\mathrm{GSH}\right]}{\left[\mathrm{PSH}\right]\left[\mathrm{GSSG}\right]}$$

For most protein cysteines, K_mix_ is approximately 1, indicating that the GSH:GSSG ratio would have to decline dramatically to drive S-glutathionylation [49]. The ratio of GSH:GSSG, however, usually remains very high inside the cytosol, even during extreme conditions of oxidative stress. This is due to the capability of most cells to increase the activity of glutathione reductase or actively transport GSSG out of the cell as a protective mechanism against oxidative stress [50]. Thus, although the thiol–disulfide exchange of a protein thiolate with GSSG may occur spontaneously, the reaction is slow due to a lack of extreme conditions. The absence of a detectable increase in GSSG during S-glutathionylation stimulated by the respiratory burst in human neutrophils provides evidence against a thiol–disulfide exchange mechanism [51]. Therefore, except for proteins, such as the transcription factor c-Jun, that display unusually high thiol redox potentials (K_mix_~13) [52], the thiol–disulfide exchange reaction is not a likely mechanism for S-glutathionylation, and proteins shown to be S-glutathionylated by GSSG in vitro may not reflect what occurs in vivo.

### 4.2. Reactive Thiol Intermediates for S-Glutathionylation

The mechanisms that are more likely to mediate S-glutathionylation reactions in vivo involve reactive thiol derivates such as sulfenic acids, sulfenyl-amides, thiyl radicals, S-nitrosylated thiols, and thiosulfinates.

#### 4.2.1. Sulfenic Acids

The two-electron oxidation of a cysteine thiolate generates a sulfenic acid, the simplest oxyacid of sulfur. The oxidants most commonly implicated in the conversion of protein thiolates to their corresponding sulfenic acids include hydrogen peroxide, alkyl hydroperoxides, peroxynitrite, hypochlorous acid, and chloramines [20]. Under prolonged exposure to these oxidants, sulfenic acids may be oxidized to more stable and irreversible thiol derivatives such as sulfinic and sulfonic acids or may react with neighboring thiols to form disulfides [20]. The abundance of GSH in cells is known to rapidly react with protein sulfenic acids to displace the hydroxyl group and form S-glutathionylated proteins [20]. It is plausible that a glutathione sulfenic acid could react by a similar mechanism with a protein thiolate to generate the S-glutathionylated protein, but the low acidity of the GSH thiol (pK_a_ = 9.6) is likely to limit this reaction in vivo [53].

Many proteins have been identified as candidates for regulation by sulfenic acid formation. However, most of these studies were performed in vitro and in the absence of GSH. Recent literature suggests that although sulfenic acid formation may be the initial oxidative modification for these proteins, S-glutathionylation serves as the more stable intermediate in redox signaling [54]. This was shown for the molecular chaperone BiP, initially suggested to be regulated by the sulfenic acid formation and later shown to undergo S-glutathionylation through a sulfenic acid intermediate [55]. Similarly, mammalian protein tyrosine phosphatases (PTPs), initially observed to react with H_2_O_2_ to form a protein sulfenic acid [56,57], have been shown to undergo S-glutathionylation in the presence of GSH [58]. More recently, Heppner et al. showed that the oxidation of epidermal growth factor receptor (EGFR) and non-receptor tyrosine kinase Src by the NADPH oxidase DUOX1 involved sequential oxidation to sulfenic acids and S-glutathionylated proteins [59]. In agreement with these findings, sulfenic acids have been shown to react much faster with GSH than with oxidants like H_2_O_2_, thereby escaping further oxidative modification [60].

#### 4.2.2. Sulfenyl-Amides

The formation of the sulfenyl-amide species was first demonstrated in vitro by Salmeen et al., as an alternative protective modification for the active site cysteine of protein tyrosine phosphatase 1B (PTP1B) [61]. The proposed mechanism involved the initial oxidation of Cys215 via H_2_O_2_ to sulfenic acid, followed by the rapid elimination of oxygen to generate a more stable derivative, the sulfenyl-amide species [61]. X-ray crystallographic analysis showed that the sulfur atom of Cys215 formed a cyclic covalent bond to the primary chain nitrogen of an adjacent residue, resulting in a conformational change in the catalytic site of the protein [61]. The sulfenyl-amide bond was fully reducible upon the addition of GSH. As earlier studies had demonstrated that PTP1B activity could be regulated by the S-glutathionylation of Cys215 [59], the sulfenyl-amide bond was proposed to serve as an intermediate to facilitate the formation of S-glutathionylated PTP1B [61]. At present, whether this mechanism is relevant in vivo or for proteins other than PTP1B remains unknown.

#### 4.2.3. Thiyl Radicals

Thiyl radicals are highly reactive species that can be generated via electron transfer or hydrogen atom abstraction from a thiol [62,63,64]. Free radical species that oxidize thiols to thiyl radicals include hydroxyl (^•^OH), carbonate (CO_3_^•–^), nitrogen dioxide (^•^NO_2_), superoxide (O_2_^•–^), and peroxyl and phenoxyl radicals [17].

The formation of protein thiyl radicals or glutathione thiyl radicals may lead to protein S-glutathionylation reactions through radical recombination or reaction of a radical with a thiolate followed by reaction with O_2_ [54]. In the presence of glutathione thiyl radical-generating systems (Fe^2+^/ADP/H_2_O_2_ + GSH or horseradish peroxidase/H_2_O_2_ + GSH), several proteins have been shown to undergo S-glutathionylation in vitro [65]. A recent study by Kang et al., shows that a burst in the mitochondrial superoxide production leads to thiyl radical formation resulting in S-glutathionylation of the mitochondrial Complex I [66,67]. Other researchers have discovered that superoxide induces endothelial nitric-oxide synthase (eNOS) protein thiyl radical formation, leading to the modification of cysteine with either disulfide bond formation or S-glutathionylation [68,69]. Thus, it seems likely that thiyl radicals may serve as regulatory intermediates leading to S-glutathionylation reactions in vivo. Moreover, there is evidence that these reactions may be catalyzed by glutaredoxin [54].

#### 4.2.4. S-Nitrosylated Thiols

S-nitrosylation is a type of post-translational modification that can occur under normal and pathological cellular conditions, analogous to S-glutathionylation [70]. It is characterized by the coupling of a nitric oxide (·NO) moiety to a reactive cysteine thiol, forming an S-nitrosothiol [71,72]. The S-nitrosothiol of glutathione (GSNO) is a relatively stable molecule found in micromolar concentrations in healthy tissues [54]. Although a variety of reagents, including nitrous acid, nitrogen dioxide and nitrosyl chloride can react with GSH to induce GSNO formation, dinitrogen trioxide (N_2_O_3_) is considered to be the primary nitrosylating agent [73]. During conditions of nitrosative stress, specific cell compartments can produce higher levels of GSNO, which can promote both the S-nitrosylation and S-glutathionylation of proteins [74,75]. Alternatively, the reaction between a protein S-nitrosothiol and glutathione may also lead to S-glutathionylation, although there is little information regarding this mechanism.

In the presence of physiologically relevant concentrations of GSH, one study showed that NO inhibits the DNA binding activity of the transcription factor c-Jun by specifically inducing S-glutathionylation in its DNA binding site [76]. Another study with isolated proteins showed that papain, creatine phosphokinase, and glyceraldehyde-3-phosphate dehydrogenase (GAPDH) were significantly both S-nitrosylated and S-glutathionylated by GSNO, whereas alcohol dehydrogenase, bovine serum albumin, and actin were only S-nitrosylated [77]. The S-nitrosylation of GAPDH was shown to be unstable and decompose spontaneously, whereas S-glutathionylation led to the inhibition of the enzyme [78]. Thus, it is plausible that S-nitrosylation and S-glutathionylation may exert opposing effects on protein function [79]. At present, the structural features that select S-glutathionylation over S-nitrosylation or vice versa remain largely uncertain but are likely related to the local microenvironment of the target cysteine.

#### 4.2.5. Thiosulfinates

Glutathione thiosulfinate (also called glutathione disulfide S-oxide, GS(O)SG) is the anhydride of glutathione sulfenic acid [80]. Li et al. identified this unique molecule as a product derived from the spontaneous decomposition of GSNO in aqueous solution [81]. The mechanism for the generation of glutathione thiosulfinate is not yet known, although it has been speculated that the initiation of GSNO decomposition may be catalyzed by copper ions through homolysis, yielding GSH and NO [73,80]. Glutathione thiosulfinate may also be generated through the oxidation of a disulfide or thiol by the O_2_^•–^/H_2_O_2_-producing system xanthine/xanthine oxidase [80,81].

Glutathione thiosulfinate is highly reactive with thiols and has been suggested to mediate S-glutathionylation of several proteins. In the rat brain, glutathione thiosulfinate was shown to be one of the most potent S-glutathionylating agent among the various glutathione derivatives tested, including GSNO and GSSG [81]. As glutathione thiosulfinate is the degradation product of GSNO, its formation may account for some of the effects of GSNO-induced S-glutathionylation [81]. The formation of the thiosulfinate product has been proposed to mediate the S-glutathionylation of rat brain neurogranin (Ng) [81], matrix metalloproteinases [82], and tyrosine hydroxylase [83]. At present, it remains unclear whether glutathione thiosulfinate or other glutathione derivatives (sulfenic acid, thiyl radical, etc.) play more critical roles as intermediates in the S-glutathionylation mechanism in vivo.

## 5. Enzymatic Protein S-Glutathionylation

Although protein S-glutathionylation in cells may involve non-enzymatic chemical reactions, several enzymes have been proposed to catalyze the transfer of glutathione to the target protein.

### 5.1. Glutathione S-Transferase π

Glutathione S-transferases (GSTs) are an abundant family of detoxification enzymes that catalyze the conjugation of glutathione to chemically reactive electrophilic compounds [84]. The Pi class of GSTs (GSTπ) specifically participates in resistance to drugs and carcinogens. High levels of GSTπ are shown to occur in solid tumors, although their function in the catalytic detoxification process remains ambiguous [85]. A link between GSTπ and S-glutathionylation was first described through the observation that GSTπ could conjugate GSH to peroxiredoxin VI, a non-selenium-dependent lipid peroxidase that converts lipid hydroperoxides to their corresponding alcohols [86].

A critical step in the detoxification reaction catalyzed by peroxiredoxin VI is the formation of the enzyme sulfenic acid on Cys47, the single conserved cysteine residue of peroxiredoxin VI [86]. Cys47 is sterically inaccessible within the enzyme, making the sulfenic acid relatively stable within the globular dimer complex [85]. In addition, the oxidized monomer of the enzyme forms a homodimer that further limits its accessibility [86]. Manevich et al. demonstrated that the heterodimerization of peroxiredoxin VI with GSH-saturated GSTπ resulted in the S-glutathionylation of the oxidized cysteine followed by the subsequent reduction of the mixed disulfide and the activation of the enzyme [86]. These results suggest that GSTπ can facilitate the transfer of GSH to the active site of peroxiredoxin VI through a physiological mechanism that restores the enzymatic activity.

GSTπ has been described to play a key role in the S-glutathionylation mechanism of several proteins, especially during oxidative stress conditions in vivo and in vitro [87,88]. Townsend et al. have shown that GSTπ knockout mice have decreased S-glutathionylated protein levels following PABA/NO treatment and that the rate of S-glutathionylation is significantly enhanced in the presence of GSTπ [85]. In agreement with these findings, in vitro studies using GSTπ-knockdown HEK293 cells have shown decreased levels of S-glutathionylation [89]. Moreover, there is evidence that GSTπ present in the endoplasmic reticulum catalyzes S-glutathionylation of critical proteins within the organelle, providing a potential linkage between redox-based signaling and pathways that regulate the unfolded protein response [90].

Recent studies have identified additional proteins that may also undergo selective S-glutathionylation by GSTπ, including Nrf2 protein inhibitor Keap1, [91], estrogen receptor α [92], actin [93], and AMP-activated protein kinase [94]. Furthermore, it is important to note that GSTπ is also subject to redox regulation [95]. Under oxidative stress, S-glutathionylation on Cys47 and Cys101 is shown to autoregulate GSTπ by oligomerization and enzyme inactivation [85]. Therefore, GSTπ may promote the S-glutathionylation of a wide range of proteins, and its high expression in tumor cells may be related to the aberrant oxidative stress state [96].

### 5.2. Other Potential Enzymes

Under normal physiological conditions with high levels of GSH, glutaredoxin (Grx) primarily functions in catalyzing the deglutathionylation reaction (i.e., removing glutathione from S-glutathionylated proteins), which is discussed in more detail below. However, during increased levels of oxidative stress, when glutathione is present mainly in its oxidized forms including GSSG, GSNO, and GS^•^, Grx1 has been proposed to catalyze the forward S-glutathionylation reaction [97]. Mieyal et al. anticipated that due to the low pK_a_ of the active site Cys22 of Grx1 and the stability of disulfide-anion radicals (i.e., Grx1- SSG^•−^) as opposed to the glutathionyl radical (GS^•^), Grx1 may catalyze the S-glutathionylation of proteins through a radical intermediate [49]. Grx1 has been shown to promote S-glutathionylation of GAPDH, actin, and PTP1B using glutathionyl radical as the proximal donor [65]. Besides, S-glutathionylation was competitively inhibited by the reaction of O_2_ with Grx1- SSG^•−^, confirming that the redox environment likely determines the mode of catalysis by Grx1 [65].

Recently, a possible role for the glyoxalase II enzyme in the S-glutathionylation of specific proteins has been reported [98,99]. The glyoxalase system, composed of glyoxalases I and II, is involved in the detoxification of methylglyoxal and other reactive aldehydes produced during metabolism [100]. Reduced glutathione is used to convert methylglyoxal to S-D-lactoylglutathione, a thioester of glutathione [100]. The product is then hydrolyzed by glyoxalase II to yield D-lactate and reduced glutathione [100]. Ercolani et al. showed that when the glyoxalase II and S-D-lactoylglutathione were incubated with malate dehydrogenase or with actin, the proteins were S-glutathionylated [98]. At present, a role for glyoxalase II for S-glutathionylation in vivo has not been reported and further investigation is needed to elucidate the potential interaction of the catalytic site of the enzyme with target proteins.

## 6. Deglutathionylation

Protein S-glutathionylation is a dynamic and reversible process, dependent on both the rate of formation and the rate of deglutathionylation. The reversibility of protein S-glutathionylation provides an additional layer of regulation for controlling cellular processes [101]. Deglutathionylation reactions in cells may occur spontaneously due to the reductive environment and high concentrations of GSH in cells. However, glutaredoxin has specific binding grooves for glutathione and is considered the prime catalyst for deglutathionylation [48]. Glutaredoxin is a member of the thioredoxin superfamily of enzymes, along with thioredoxins, protein disulfide isomerase, glutathione peroxidase, and glutathione S-transferase [102]. Although these proteins show low similarity in their sequence homology, they all incorporate the thioredoxin fold structural motif, which is essential for their redox function [102].

Isoforms of glutaredoxin are found in prokaryotes and the various cellular compartments of eukaryotes. Based on their active site sequences, mammalian glutaredoxins can be broadly categorized into two major subfamilies: class I and class II glutaredoxins [103]. Class I glutaredoxins are localized in the cytosol and mitochondrial intermembrane space, whereas class II glutaredoxins are primarily localized in mitochondria. Class I glutaredoxins are better characterized and have been reported to catalyze most of the deglutathionylating activity in mammalian cells [104,105]. Class II glutaredoxins bind Fe-S clusters and generally exhibit lower oxidoreductase activity than that of class I glutaredoxins [106,107].

**Figure 3:**
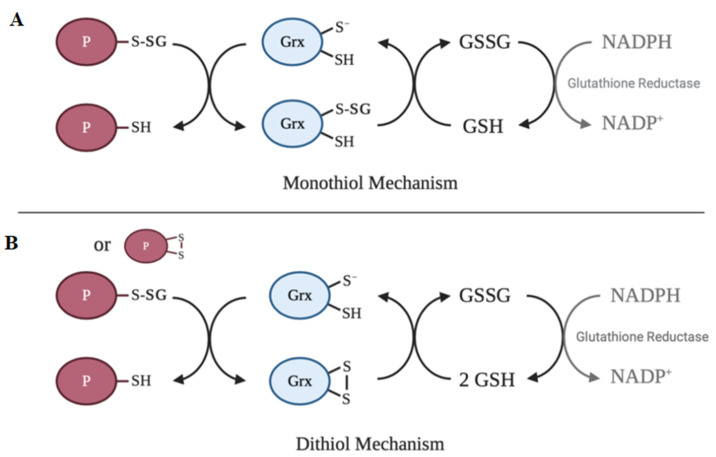
Catalytic mechanisms of deglutathionylation by glutaredoxin. (**A**) In the monothiol mechanism of deglutathionylation, the thiolate anion of glutaredoxin attacks the S-glutathionylated protein thiol, forming an enzyme-intermediate and releasing the reduced protein. The second step in this mechanism involves the reduction of the enzyme intermediate by GSH to produce GSSG, which is subsequently reduced by glutathione reductase and NADPH. (**B**) The dithiol mechanism uses both active site cysteine residues of glutaredoxin to reduce glutathionylated substrates. Oxidized glutaredoxin is reduced in the presence of 2 GSH molecules to regenerate the active enzyme.

The active site of the vast majority of glutaredoxin isoforms contain variations of the redox-active Cys-X-X-Cys motif [108]. The main activity of glutaredoxin involves thiol–disulfide exchange and nucleophilic displacement reactions. A monothiol mechanism and a dithiol mechanism have been proposed, but the monothiol mechanism is generally considered to be the prevalent deglutathionylation mechanism [49]. In the monothiol mechanism, the S-glutathionylated protein thiol is attacked by the thiolate anion of glutaredoxin, forming an enzyme intermediate (Grx-SSG) and releasing the reduced protein. The second step in this mechanism involves the reduction of the enzyme intermediate by GSH to produce GSSG, which is subsequently reduced by glutathione reductase and NADPH [49]. In contrast, the dithiol mechanism uses both active site cysteine residues of glutaredoxin to reduce glutathionylated substrates. Figure 3 depicts a summary of the catalytic mechanism of deglutathionylation by glutaredoxin.

Another enzyme that has been proposed to have deglutathionylating activity is sulfiredoxin (Srx), a small oxidoreductase that catalyzes the reduction of sulfinic acid derivatives of 2-Cys peroxiredoxins [109]. Findlay et al. observed that the overexpression of Srx in HEK293 cells lowered the levels of S-glutathionylated proteins, such as actin and PTP1B, induced by the diazeniumdiolate PABA/NO [110,111]. Furthermore, Park et al. showed that Srx is specific for deglutathionylating Prx I (known to have a high affinity to Srx) and that the level of S-glutathionylated Prx I was substantially elevated in Srx-knockdown cells [109]. The S-glutathionylation of Prx V, not known to bind to Srx, was not altered by Srx expression levels, demonstrating the specificity of Srx towards Prx I [109]. Although these studies demonstrate that Srx may contribute to the catalysis of deglutathionylation, whether the contribution is significant in vivo is unclear.

## 7. Structure–Function Relationship of Protein S-Glutathionylation

S-glutathionylation can directly regulate the structure/function of a wide range of proteins, and thereby transduce redox signals in cellular pathways. S-glutathionylation has been shown to either increase or decrease the activity of several classes of proteins including mitochondrial enzymes, heat shock proteins, transcription factors and cytoskeletal proteins [32]. In contrast to the various studies that focus on the functional aspects of S-glutathionylation (Table 1), very few have identified specific modification sites and the structural changes in the modified proteins.

One study demonstrates how S-glutathionylation can alter the structure and regulate the function of hHsp70, a cytosolic stress-induced form of heat shock protein 70 (Hsp70), in HeLa cells [117]. Hsp70 proteins are molecular chaperones involved in maintaining protein homeostasis by facilitating protein folding, directing misfolded proteins to the cellular degradation machinery, and preventing protein aggregation [118]. The function of hHsp70 relies on the allosteric communication between two individual domains of the enzyme, namely the ATPase or nucleotide-binding domain (NBD) and the substrate-binding domain (SBD). The allosteric mechanism couples the ATP hydrolysis cycle in the NBD with the binding and release of the substrate polypeptides [118]. Cys574 and Cys603, located in the C-terminal α-helical lid of the SBD, were shown to undergo S-glutathionylation both in the presence and absence of nucleotide-binding [117]. The same study showed that the S-glutathionylation of these residues resulted in the unfolding of the α-helical lid structure. This unfolded region was shown to bind to and block the substrate-binding site, thereby mimicking the substrate and leading to an increase in the ATPase activity of the enzyme and a subsequent decrease in the binding ability of external substrates, including heat shock transcription factor 1 (Hsf1) [117]. Following deglutathionylation of the cysteine residues by treatment with DTT, the conformational change in the SBD was shown to be fully reversible [117].

Another study shows how S-glutathionylation of the binding immunoglobulin protein (BiP) regulates the balance between its foldase and ATPase activities by altering protein structure [119]. BiP is another Hsp70 molecular chaperone localized in the endoplasmic reticulum. It is an essential component of the translocation machinery for newly synthesized proteins and plays a primary role in the activation of the unfolded protein response [120]. Upon stimulation with disulfiram, S-glutathionylation was shown in Cys41 and Cys420 [119]. Using circular dichroism spectroscopy, the secondary structure of S-glutathionylated BiP was compared to that of the native form of the protein revealing a decreased α-helix content and increased β-sheets. These changes corresponded to a decrease in the ATPase activity of S-glutathionylated BiP and an increase in the foldase activity [119]. Studies using recombinant BiP protein with mutations from Cys to Ala showed that while Cys41 is important for ATPase activity, Cys420 is important for foldase activity [119]. Thus, the structural changes which lead to enhanced foldase activity allow BiP to maintain more accurate protein folding during oxidized conditions in the endoplasmic reticulum [119]. These observations highlight how local structural changes associated with S-glutathionylation can cause global effects in altering the function of Hsp70 proteins.

## 8. Implications of Protein S-Glutathionylation in Diseases

A large body of evidence has accumulated over the past few decades suggesting that oxidative stress may be involved, with varying degrees of importance, in the pathogenesis of diseases [121]. In many models of disease, an increased level of protein S-glutathionylation associated with oxidative stress has been shown [122,123,124,125,126,127]. Moreover, S-glutathionylated proteins have been investigated as potential biomarkers of human disease [128,129,130].

Here, we discuss recent studies that have associated a number of conditions such as aging, cardiovascular disease, cancer, and liver disease with changing levels of S-glutathionylation.

### 8.1. Aging and Neurodegeneration

The aging process is a complex phenomenon broadly defined as the progressive decline in physiological functions accompanied by a wide variety of pathological conditions [131]. Several theories that explain the aging process have been proposed, including the oxidative stress theory, which proposes that elevated levels of ROS that accompany aging lead to functional alterations in cellular macromolecules [132]. In addition to elevated ROS production, one study by Zhu et al., suggests a significant reduction in the GSH levels as well as the GSH:GSSG ratio in the brains of aged rats [133].

Aging is considered to be the biggest risk factor for most neurodegenerative diseases. Neurodegenerative diseases involve loss of neurons, aggregation of misfolded proteins, mitochondrial dysfunction, and overexposure to ROS [134]. Due to increased levels of oxidative stress, it has been suggested that S-glutathionylation of specific proteins may contribute to the progression of neurogenerative diseases [134]. Several target proteins of S-glutathionylation in neurodegenerative diseases have been described previously [49,134].

The major neurodegenerative disease known to affect the aging population is Alzheimer’s disease (AD). Although the exact causes of AD are not fully understood, the loss of synapses and the accumulation of plaques formed by the protein amyloid beta, and neurofibrillary tangles formed by the protein tau are widely studied [135]. Implicated in these changes is the elevated generation of ROS, as shown by the significant extent of oxidative damage in the brains of patients suffering from AD [136]. In a postmortem AD inferior parietal lobule, Newman et al. identified deoxyhemoglobin, α-crystallin B, glyceraldehyde 3-phosphate dehydrogenase (GAPDH), and α-enolase to be sensitive to S-glutathionylation [137]. The same study showed that S-glutathionylated GAPDH and α-enolase have reduced activity [137]. Brain tissues from AD patients revealed the selective S-glutathionylation of the monomeric and dimeric form of the proapoptotic protein, p53 [138]. The authors proposed that the S-glutathionylation of p53 may prevent the formation of the tetramer, which is the active conformation of the protein [138]. To further support these findings, a recent study showed that the plasma GSH/GSSG ratio was significantly reduced in AD patients, and this altered redox status could contribute to the S-glutathionylation of proteins [139].

### 8.2. Cardiovascular Disease

Mitochondria are one of the most important sources of ROS in the cell. Mitochondria are abundant in cardiac cells due to the increased energy demands of producing ATP through coupled electron transport and oxidative phosphorylation [140]. Therefore, mitochondrial dysregulation and increased ROS production are thought to contribute significantly to cardiac pathology [141]. Although a role for oxidative stress is not yet established, increased production of ROS indicates the likelihood of redox regulation by S-glutathionylation in cardiovascular diseases [142].

#### 8.2.1. Myocardial Infarction

Myocardial infarction, also known as a heart attack, occurs due to the formation of plaques in the interior walls of the arteries, which block blood flow and reduce oxygen supply to the cardiac muscle [143]. Myocardial infarction results in contractile dysfunction, increased inflammation, and apoptosis [143]. Due to increased levels of oxidative stress, several proteins responsible for maintaining the contractility of the heart may be susceptible to undergo S-glutathionylation. One study showed that ischemia-reperfusion injury (IR) induced a 15-fold increase in the S-glutathionylation of the cardiac proteome [144]. In a rat model of in vivo IR, Chen et al. provided evidence for the DTT-reversible S-glutathionylation of actin [145]. Consistent with previously published data, S-glutathionylation decreased the efficiency of actin polymerization and reduced its cooperativity in binding tropomyosin [145]. This finding indicates that the modification of the protein may contribute to the function of contractile filaments during IR injury. In contrast to actin, mitochondrial complex II was shown to undergo deglutathionylation accompanied by a marked loss of the enzyme activity following IR [146].

A more recent study shows an increase in the S-glutathionylation of a high-molecular-weight protein (most likely titin) in the early postmyocardial infarction period in mouse hearts [147]. Titin is a giant elastic protein expressed in the sarcomeres of cardiomyocytes, whereas actin and myosin generate force by the sliding-filament mechanism, titin generates “passive” tension and contributes to the myocardial wall stiffness. The mechanical unfolding of titin immunoglobulin domains exposes buried cysteines that may undergo S-glutathionylation [148]. The S-glutathionylation of these cryptic cysteines decreases the refolding of the protein and increases the elasticity of cardiomyocytes [148]. This effect is reversed upon the introduction of reducing conditions [148]. Thus, the S-glutathionylation of titin may represent a general mechanism to regulate tissue elasticity.

Another protein involved in the regulation of cardiac dynamics is cardiac myosin binding protein C (cMyBP-C). One study showed that a decrease in the GSH/GSSG ratio in isolated perfused rat hearts led to increased S-glutathionylation and reduced protein kinase A (PKA)-dependent phosphorylation of cMyBP-C [149]. The physiological relevance of this observation was determined in vivo in the context of physical exercise [149]. Moderate exercise increased S-glutathionylation and PKA-dependent phosphorylation of cMyBP-C and had a positive effect on systolic cardiac function [149]. On the other hand, higher cardiac stress induced by prolonged exercise increased cMyBP-C S-glutathionylation, but reduced PKA-dependent phosphorylation and led to cardiac dysfunction [149]. These results indicate crosstalk between S-glutathionylation and phosphorylation of cardiac contractile regulatory proteins.

Ischemic preconditioning refers to brief, repetitive periods of ischemia that make the myocardium more resistant to larger ischemic insult and thus confer protection against subsequent myocardial infarction [150]. Preconditioning of the myocardium by electrically induced tachycardia increases the activity of the ryanodine receptor 2 (RyR2), a channel that controls the flow of Ca^2+^ out of the sarcoplasmic reticulum in cardiac muscle [151]. Several studies have linked myocardial infarction to increased RyR2 activity in response to oxidative stress [152]. Sanchez et al. show that preconditioning by tachycardia induces an increase in ROS generation through NOX activation in SR-enriched microsomal fractions isolated from the canine ventricle [116]. Subsequently, this led to an increase in S-glutathionylation of RyR2 [116]. Although the specific mechanisms of RyR2 modification by S-glutathionylation remain to be elucidated, in vitro studies suggest that the S-glutathionylation of RyR2 during preconditioning may have a protective role by increasing systolic Ca^2+^ release and decreasing diastolic Ca^2+^ leak [116,153].

#### 8.2.2. Cardiac Hypertrophy

Cardiac hypertrophy refers to the enlargement and thickening of cardiomyocytes. It is classified as physiological when associated with normal cardiac function or pathological when associated with cardiac dysfunction [154]. Pathological hypertrophy is associated with cell death, fibrosis, dysregulation of Ca^2+^-handling proteins, as well as mitochondrial dysfunction, and eventually progresses to heart failure [155]. Among the various signaling pathways that contribute to pathological cardiac hypertrophy, the Raf/MEK/ERK pathway plays a central role in mediating cardiomyocyte growth [156,157]. Pimental et al. demonstrated that the Raf/MEK/ERK pathway stimulated by mechanical stretch in neonatal rat ventricular myocytes is dependent on the S-glutathionylation of p21Ras, a low molecular mass GTPase implicated in myocyte growth signaling [158]. The mechanical strain-induced S-glutathionylation of p21Ras was inhibited by catalase and Grx overexpression and reversed by the addition of DTT [158]. Likewise, Adachi et al. previously demonstrated that S-glutathionylation of p21Ras regulates angiotensin II-induced hypertrophy in vascular smooth muscle cells [159]. Collectively, these observations suggest that the S-glutathionylation of p21Ras is involved in the transduction of strain-stimulated cardiac hypertrophy [158] via activation of the Raf/MEK/ERK growth pathway.

### 8.3. Cancer

Cellular antioxidant defense mechanisms minimize the harmful effects of oxidative stress and maintain the optimal redox environment under normal physiological conditions. However, cancer cells differ from normal cells as they exhibit high ROS levels due to genetic, metabolic, and microenvironment-associated alterations and mitochondrial dysfunction [160]. To prevent damage from excessive ROS production, cancer cells respond to oxidative stress by adjusting their antioxidant enzymes and using metabolic pathways to provide higher levels of antioxidant molecules, such as GSH and NADPH [161]. Based on these observations, disabling antioxidant mechanisms by promoting ROS production has been investigated as a potential anticancer strategy [161].

Numerous studies have reported that S-glutathionylation can modify proteins involved in the signaling mechanisms of cancer cells. In particular, protein kinase C (PKC) isozymes, which link multiple cellular processes to cancer, contain cysteine-rich regions that are highly susceptible to oxidants [162,163]. PKC isozymes are involved in the expression of genes relevant for cell cycle progression, tumorigenesis, and metastatic dissemination [162]. In a study by Chu et al., the thiol-specific oxidant diamide was shown to induce potent GSH-dependent inactivation of several PKC isozymes via S-glutathionylation [164]. Similarly, Cys199 in the activation loop of PKCα is susceptible to undergo S-glutathionylation, resulting in the complete inactivation of the enzyme and dephosphorylation of threonine-197 in PC12 cells [165,166]. In addition to PKC isozymes, S-glutathionylation has been shown to inactivate the phosphatases of the PI3-kinase-Akt pathway, which plays a critical role in cell survival and proliferation [167,168].

Many transcription factors have conserved cysteine residues that are susceptible to thiol modifications. S-glutathionylation of these transcription factors may interfere with their ability to bind to DNA, thereby dysregulating gene expression. For instance, the tumor suppressor p53, known to play an essential role in cell cycle control and apoptosis, is inhibited by the S-glutathionylation of cysteines present within its proximal DNA-binding domain [169]. Untreated cells showed low levels of S-glutathionylated p53, which increased significantly after oxidant or anticancer drug (camptothecin or cisplatin) treatment [169]. Other transcription factors shown to lose their DNA-binding activity by S-glutathionylation include NF-κB and c-Jun, and both play a critical role in the pathogenesis of cancer [170,171]. Furthermore, the S-glutathionylation of transcription factors, such as STAT3, that promote cell survival and proliferation may have important implications for the development of new anticancer drugs [172].

Cancer is a disease of uncontrolled cell growth that occurs by the overactivation of proliferation and corresponding inhibition of apoptosis. Thus, reinstating apoptosis in malignant cells can lead to the selective elimination of cancer cells [173]. Caspases are a family of cysteine proteases that play a central role in the execution of apoptosis and may be targeted to develop therapeutic strategies in human cancers [174,175]. In vitro experiments have shown that S-glutathionylation of procaspase-3 at Cys145 of the p17 subunit and Cys45 of the p12 subunit leads to the inhibition of caspase-3 activation and activity [176]. Furthermore, TNF-induced apoptosis was shown to be accompanied by an increase in Grx activity and deglutathionylation of caspase-3 [177]. Cysteine-to-serine mutations of caspase-3 prevented S-glutathionylation and resulted in increased cleavage compared to wild-type caspase-3 [177]. Recently, we have shown that caspase-3 activation in staurosporine-induced apoptosis is concurrent with the depletion of cellular glutathione and the generation of ROS, supporting the notion that the cellular redox environment may be a critical factor for the activation of the protease [178]. Although these studies indicate that S-glutathionylation may be involved in the regulation of caspase activity, whether an analogous mechanism is applicable for cancer cells in vivo remains to be discovered.

### 8.4. Liver Disease

Nonalcoholic fatty liver disease (NAFLD) is a common hepatic condition that encompasses a wide variety of liver diseases characterized by the accumulation of excess fat stored in liver cells [179]. Although the overall disease progression of NAFLD has been described, the mechanisms underlying its initiation and progression remain poorly understood [180]. In combination with other contributors like insulin resistance, oxidative stress appears to be one of the significant mechanisms in the pathogenesis of NAFLD and in the progression from steatosis (accumulation of triglyceride inside hepatocytes) to steatohepatitis, which is an advanced form of liver disease defined by the presence of steatosis along with hepatocyte death [181].

Recent studies have shown that mice fed with a high-fat diet develop NAFLD and show increased S-glutathionylation [48,182]. In particular, sirtuin-1 (SirT1), a central metabolic regulator and NAD^+^-dependent histone deacetylase, has been identified as a target protein for increased S-glutathionylation [112]. S-glutathionylation of SirT1 inactivates the enzyme and modulates lipid metabolism via hyperacetylation of key transcription factors, such as p53 and sterol regulatory element-binding protein (SREBP) [112]. The activation of p53 occurs in response to cellular stress and is an essential regulator of the cell cycle, senescence, and apoptosis [183]. Under high oxidative stress conditions, hepatocytes may utilize p53 to initiate apoptosis involving p53 upregulated modulator of apoptosis (PUMA), which modulates apoptosis via interaction with the antiapoptotic protein Bcl-2 [184]. The overexpression of Grx restores SirT1 activity and prevents the activation of p53 [112]. In agreement with these findings, the overexpression of SirT1 has been shown to improve NAFLD [185] and conversely, SirT1 knockout mice have been shown to develop NAFLD [186].

In another study, S-glutathionylation was associated with TNF α-mediated cytotoxicity [182]. The proinflammatory cytokine tumor necrosis factor-α (TNF α) has been suggested to play a central role in the pathogenesis of NAFLD [187,188]. TNFα activates nuclear factor-kappa B (NF-κB) signaling pathways to induce transcription of target genes that primarily encode survival proteins [189,190]. Dou et al. have shown that mice fed with a high-fat diet develop NAFLD and show increased protein S-glutathionylation in the liver [182]. The accumulation of GSSG was shown to prevent TNF α-induced activation of IKK-β, an upstream kinase in the NF-κB signaling pathway, by inducing IKK-β S-glutathionylation [182]. This was confirmed in animal studies showing that high-fat diet consumption resulted in increased hepatic IKK-β S-glutathionylation, leading to suppressed IKK-β activation and subsequent NF-κB suppression [182]. Moreover, the same study showed that high-fat diet consumption led to a decreased hepatic expression of Grx, suggesting that the observed increase in S-glutathionylated protein formation may occur as a result of both increased S-glutathionylation and suppressed deglutathionylation reactions.

## 9. Concluding Remarks

Over the past few decades, the oxidative post-translational modifications of protein cysteine residues have emerged as an attractive area of research in redox biochemistry. In this regard, protein S-glutathionylation appears to be the predominant modification due to the abundance of the endogenous glutathione pool. S-glutathionylation serves both to protect proteins from oxidative damage and modify structure/function relationships. Increasing evidence shows that redox regulation by S-glutathionylation contributes to physiological processes, and aberrant S-glutathionylation is associated with various health conditions, from cancer to cardiovascular disease.

Despite the vast body of accumulated research in this field, much work is still needed to further our understanding of this unique protein modification. As we have discussed in this review, multiple cellular mechanisms of S-glutathionylation have been proposed. However, the contributions of these mechanisms in experimental models remain uncertain, and the functional implications of the enzyme-mediated S-glutathionylation in physiological settings are unclear. Further investigation into the mechanisms of protein S-glutathionylation and the identification of the changes in S-glutathionylation patterns in disease contexts will provide more insight into the significance of S-glutathionylation as a fundamental post-translational modification.

## Figures and Tables

**Table 1:** Examples of the effect of S-glutathionylation on protein function.

Protein	Reported Effect of S-Glutathionylation on Function	References
c-Jun	Inhibition	[52]
Protein tyrosine phosphatase 1B (PTP1B)	Inhibition	[58]
Glyceraldehyde-3-phosphate dehydrogenase (GAPDH)	Inhibition	[78]
Estrogen receptor α	Inhibition	[92]
AMP-activated protein kinase (AMPK)	Activation	[94]
Sirtuin-1	Inhibition	[112]
Antigen 85C *	Inhibition	[113]
Transcriptional Co-activator with PDZ-binding Motif (TAZ)	Activation	[114]
Catalase	Inhibition	[115]
Ryanodine receptor 2 (RyR2)	Activation	[116]

* indicates a bacterial protein.

## Abbreviations

ROS	reactive oxygen species
GSH	reduced glutathione
GSSG	oxidized glutathione
RSSR’	disulfide
RSOH	sulfenic acid
RSO_2_H	sulfinic acid
RSO_3_H	sulfonic acid
RSNHR’	sulfenyl-amide
GGT	γ-glutamyl transpeptidase
GST	glutathione S-transferase
Grx	glutaredoxin

## References

[1] Sies H., Jones D.P. (2020). Reactive oxygen species (ROS) as pleiotropic physiological signalling agents. Nat. Rev. Mol. Cell Biol..

[2] Panday A., Sahoo M.K., Osorio D., Batra S. (2015). NADPH oxidases: An overview from structure to innate immunity-associated pathologies. Cell. Mol. Immunol..

[3] Liguori I., Russo G., Curcio F., Bulli G., Aran L., Della-Morte D., Gargiulo G., Testa G., Cacciatore F., Bonaduce D. (2018). Oxidative stress, aging, and diseases. Clin. Interv. Aging.

[4] Finkel T. (2011). Signal transduction by reactive oxygen species. J. Cell Biol..

[5] Sies H. (2017). Hydrogen peroxide as a central redox signaling molecule in physiological oxidative stress: Oxidative eustress. Redox Biol..

[6] Moran L.K., Gutteridge J.M., Quinlan G.J. (2001). Thiols in cellular redox signalling and control. Curr. Med. Chem..

[7] Leonberg A.K., Chai Y.C. (2007). The functional role of cysteine residues for c-Abl kinase activity. Mol. Cell. Biochem..

[8] Paulsen C.E., Carroll K.S. (2013). Cysteine-mediated redox signaling: Chemistry, biology, and tools for discovery. Chem. Rev..

[9] Mannaa A., Hanisch F.G. (2020). Redox Proteomes in Human Physiology and Disease Mechanisms. J. Proteome Res..

[10] Poole L.B. (2015). The basics of thiols and cysteines in redox biology and chemistry. Free Radic. Biol. Med..

[11] Marino S.M., Gladyshev V.N. (2012). Analysis and functional prediction of reactive cysteine residues. J. Biol. Chem..

[12] Pace N.J., Weerapana E. (2014). A competitive chemical-proteomic platform to identify zinc-binding cysteines. ACS Chem. Biol..

[13] Reddie K.G., Carroll K.S. (2008). Expanding the functional diversity of proteins through cysteine oxidation. Curr. Opin. Chem. Biol..

[14] Go Y.M., Chandler J.D., Jones D.P. (2015). The cysteine proteome. Free Radic. Biol. Med..

[15] Requejo R., Hurd T.R., Costa N.J., Murphy M.P. (2010). Cysteine residues exposed on protein surfaces are the dominant intramitochondrial thiol and may protect against oxidative damage. FEBS J..

[16] Ferrer-Sueta G., Manta B., Botti H., Radi R., Trujillo M., Denicola A. (2011). Factors affecting protein thiol reactivity and specificity in peroxide reduction. Chem. Res. Toxicol..

[17] Trujillo M., Alvarez B., Radi R. (2016). One-and two-electron oxidation of thiols: Mechanisms, kinetics and biological fates. Free Radic. Res..

[18] Licht S., Gerfen G.J., Stubbe J. (1996). Thiyl radicals in ribonucleotide reductases. Science.

[19] Lo Conte M., Carroll K.S. (2013). The redox biochemistry of protein sulfenylation and sulfinylation. J. Biol. Chem..

[20] Gupta V., Carroll K.S. (2014). Sulfenic acid chemistry, detection and cellular lifetime. Biochim. Biophys. Acta.

[21] Flohé L. (2016). The impact of thiol peroxidases on redox regulation. Free Radic. Res..

[22] Ziegler D.M. (1985). Role of reversible oxidation-reduction of enzyme thiols-disulfides in metabolic regulation. Annu. Rev. Biochem..

[23] Biteau B., Labarre J., Toledano M.B. (2003). ATP-dependent reduction of cysteine-sulphinic acid by, S. cerevisiae sulphiredoxin. Nature.

[24] Leichert L.I., Gehrke F., Gudiseva H.V., Blackwell T., Ilbert M., Walker A.K., Strahler J.R., Andrews P.C., Jakob U. (2008). Quantifying changes in the thiol redox proteome upon oxidative stress in vivo. Proc. Natl. Acad. Sci. USA.

[25] Le Moan N., Clement G., Le Maout S., Tacnet F., Toledano M.B. (2006). The Saccharomyces cerevisiae proteome of oxidized protein thiols: Contrasted functions for the thioredoxin and glutathione pathways. J. Biol. Chem..

[26] Jones D.P. (2008). Radical-free biology of oxidative stress. Am. J. Physiol. Cell Physiol..

[27] Held J.M., Gibson B.W. (2012). Regulatory control or oxidative damage? Proteomic approaches to interrogate the role of cysteine oxidation status in biological processes. Mol. Cell. Proteom..

[28] Winterbourn C.C., Metodiewa D. (1999). Reactivity of biologically important thiol compounds with superoxide and hydrogen peroxide. Free Radic. Biol. Med..

[29] Winterbourn C.C., Hampton M.B. (2008). Thiol chemistry and specificity in redox signaling. Free Radic. Biol. Med..

[30] Hall A., Parsonage D., Poole L.B., Karplus P.A. (2010). Structural evidence that peroxiredoxin catalytic power is based on transition-state stabilization. J. Mol. Biol..

[31] Allen E.M., Mieyal J.J. (2012). Protein-thiol oxidation and cell death: Regulatory role of glutaredoxins. Antioxid. Redox Signal..

[32] Zhang J., Ye Z.W., Singh S., Townsend D.M., Tew K.D. (2018). An evolving understanding of the S-glutathionylation cycle in pathways of redox regulation. Free Radic. Biol. Med..

[33] Ulrich K., Jakob U. (2019). The role of thiols in antioxidant systems. Free Radic. Biol. Med..

[34] Van Laer K., Hamilton C.J., Messens J. (2013). Low-molecular-weight thiols in thiol-disulfide exchange. Antioxid. Redox Signal..

[35] Gout I. (2019). Coenzyme A: A protective thiol in bacterial antioxidant defence. Biochem. Soc. Trans..

[36] Newton G.L., Rawat M., La Clair J.J., Jothivasan V.K., Budiarto T., Hamilton C.J., Claiborne A., Helmann J.D., Fahey R.C. (2009). Bacillithiol is an antioxidant thiol produced in Bacilli. Nat. Chem. Biol..

[37] Newton G.L., Buchmeier N., Fahey R.C. (2008). Biosynthesis and functions of mycothiol, the unique protective thiol of Actinobacteria. Microbiol. Mol. Biol. Rev..

[38] Manta B., Bonilla M., Fiestas L., Sturlese M., Salinas G., Bellanda M., Comini M.A. (2018). Polyamine-Based Thiols in Trypanosomatids: Evolution, Protein Structural Adaptations, and Biological Functions. Antioxid. Redox Signal..

[39] Lu S.C. (2013). Glutathione synthesis. Biochim. Biophys. Acta.

[40] Crawford R.R., Prescott E.T., Sylvester C.F., Higdon A.N., Shan J., Kilberg M.S., Mungrue I.N. (2015). Human CHAC1 Protein Degrades Glutathione, and mRNA Induction Is Regulated by the Transcription Factors ATF4 and ATF3 and a Bipartite ATF/CRE Regulatory Element. J. Biol. Chem..

[41] Kumar A., Tikoo S., Maity S., Sengupta S., Sengupta S., Kaur A., Bachhawat A.K. (2012). Mammalian proapoptotic factor ChaC1 and its homologues function as γ-glutamyl cyclotransferases acting specifically on glutathione. EMBO Rep..

[42] Tsunoda S., Avezov E., Zyryanova A., Konno T., Mendes-Silva L., Melo E.P., Ron D. (2014). Intact protein folding in the glutathione-depleted endoplasmic reticulum implicates alternative protein thiol reductants. Elife.

[43] Forman H.J., Zhang H., Rinna A. (2009). Glutathione: Overview of its protective roles, measurement, and biosynthesis. Mol. Asp. Med..

[44] Hwang C., Sinskey A.J., Lodish H.F. (1992). Oxidized redox state of glutathione in the endoplasmic reticulum. Science.

[45] Pirie N.W., Pinhey K.G. (1929). The Titration Curve of Glutathione. J. Biol. Chem..

[46] Vaish S., Gupta D., Mehrotra R., Mehrotra S., Basantani M.K. (2020). Glutathione S-transferase: A versatile protein family. 3 Biotech..

[47] Molavian H., Madani Tonekaboni A., Kohandel M., Sivaloganathan S. (2015). The Synergetic Coupling among the Cellular Antioxidants Glutathione Peroxidase/Peroxiredoxin and Other Antioxidants and its Effect on the Concentration of H_2_O_2_. Sci. Rep..

[48] Matsui R., Ferran B., Oh A., Croteau D., Shao D., Han J., Pimentel D.R., Bachschmid M.M. (2020). Redox Regulation *via* Glutaredoxin-1 and Protein *S*-Glutathionylation. Antioxid. Redox Signal..

[49] Mieyal J.J., Gallogly M.M., Qanungo S., Sabens E.A., Shelton M.D. (2008). Molecular mechanisms and clinical implications of reversible protein S-glutathionylation. Antioxid. Redox Signal..

[50] Dalle-Donne I., Milzani A., Gagliano N., Colombo R., Giustarini D., Rossi R. (2008). Molecular mechanisms and potential clinical significance of S-glutathionylation. Antioxid. Redox Signal..

[51] Chai Y.C., Ashraf S.S., Rokutan K., Johnston R.B., Thomas J.A. (1994). S-thiolation of individual human neutrophil proteins including actin by stimulation of the respiratory burst: Evidence against a role for glutathione disulfide. Arch. Biochem. Biophys..

[52] Klatt P., Molina E.P., De Lacoba M.G., Padilla C.A., Martinez-Galesteo E., Barcena J.A., Lamas S. (1999). Redox regulation of c-Jun DNA binding by reversible S-glutathiolation. FASEB J..

[53] Zaffagnini M., Bedhomme M., Marchand C.H., Morisse S., Trost P., Lemaire S.D. (2012). Redox regulation in photosynthetic organisms: Focus on glutathionylation. Antioxid. Redox Signal..

[54] Gallogly M.M., Mieyal J.J. (2007). Mechanisms of reversible protein glutathionylation in redox signaling and oxidative stress. Curr. Opin. Pharmacol..

[55] Wang J., Sevier C.S. (2016). Formation and Reversibility of BiP Protein Cysteine Oxidation Facilitate Cell Survival during and post Oxidative Stress. J. Biol. Chem..

[56] Lee S.R., Kwon K.S., Kim S.R., Rhee S.G. (1998). Reversible inactivation of protein-tyrosine phosphatase 1B in A431 cells stimulated with epidermal growth factor. J. Biol. Chem..

[57] Denu J.M., Tanner K.G. (1998). Specific and reversible inactivation of protein tyrosine phosphatases by hydrogen peroxide: Evidence for a sulfenic acid intermediate and implications for redox regulation. Biochemistry.

[58] Barrett W.C., DeGnore J.P., König S., Fales H.M., Keng Y.F., Zhang Z.Y., Yim M.B., Chock P.B. (1999). Regulation of PTP1B via glutathionylation of the active site cysteine 215. Biochemistry.

[59] Heppner D.E., Hristova M., Dustin C.M., Danyal K., Habibovic A., van der Vliet A. (2016). The NADPH Oxidases DUOX1 and NOX2 Play Distinct Roles in Redox Regulation of Epidermal Growth Factor Receptor Signaling. J. Biol. Chem..

[60] Zaffagnini M., Marchand C.H., Malferrari M., Murail S., Bonacchi S., Genovese D., Montalti M., Venturoli G., Falini G., Baaden M. (2019). Glutathionylation primes soluble glyceraldehyde-3-phosphate dehydrogenase for late collapse into insoluble aggregates. Proc. Natl. Acad. Sci. USA.

[61] Salmeen A., Andersen J.N., Myers M.P., Meng T.C., Hinks J.A., Tonks N.K., Barford D. (2003). Redox regulation of protein tyrosine phosphatase 1B involves a sulphenyl-amide intermediate. Nature.

[62] Stoyanovsky D.A., Maeda A., Atkins J.L., Kagan V.E. (2011). Assessments of thiyl radicals in biosystems: Difficulties and new applications. Anal Chem..

[63] Frey P.A. (2001). Radical mechanisms of enzymatic catalysis. Annu. Rev. Biochem..

[64] Schöneich C. (2016). Thiyl radicals and induction of protein degradation. Free Radic. Res..

[65] Starke D.W., Chock P.B., Mieyal J.J. (2003). Glutathione-thiyl radical scavenging and transferase properties of human glutaredoxin (thioltransferase). Potential role in redox signal transduction. J. Biol. Chem..

[66] Kang P.T., Zhang L., Chen C.L., Chen J., Green K.B., Chen Y.R. (2012). Protein thiyl radical mediates S-glutathionylation of complex, I. Free Radic. Biol. Med..

[67] Kang P.T., Chen C.L., Chen Y.R. (2015). Increased mitochondrial prooxidant activity mediates up-regulation of Complex I S-glutathionylation via protein thiyl radical in the murine heart of eNOS(-/-). Free Radic. Biol. Med..

[68] Chen C.A., Lin C.H., Druhan L.J., Wang T.Y., Chen Y.R., Zweier J.L. (2011). Superoxide induces endothelial nitric-oxide synthase protein thiyl radical formation, a novel mechanism regulating eNOS function and coupling. J. Biol. Chem..

[69] Zweier J.L., Chen C.A., Druhan L.J. (2011). S-glutathionylation reshapes our understanding of endothelial nitric oxide synthase uncoupling and nitric oxide/reactive oxygen species-mediated signaling. Antioxid. Redox Signal..

[70] Foster M.W., Hess D.T., Stamler J.S. (2009). Protein S-nitrosylation in health and disease: A current perspective. Trends Mol. Med..

[71] Ehrenfeld P., Cordova F., Duran W.N., Sanchez F.A. (2019). S-nitrosylation and its role in breast cancer angiogenesis and metastasis. Nitric Oxide.

[72] Stomberski C.T., Hess D.T., Stamler J.S. (2019). Protein S-Nitrosylation: Determinants of Specificity and Enzymatic Regulation of S-Nitrosothiol-Based Signaling. Antioxid. Redox Signal..

[73] Williams D.L.H. (1999). The Chemistry of S-Nitrosothiols. Acc. Chem. Res..

[74] Grek C.L., Zhang J., Manevich Y., Townsend D.M., Tew K.D. (2013). Causes and consequences of cysteine S-glutathionylation. J. Biol. Chem..

[75] Belcastro E., Gaucher C., Corti A., Leroy P., Lartaud I., Pompella A. (2017). Regulation of protein function by S-nitrosation and S-glutathionylation: Processes and targets in cardiovascular pathophysiology. Biol. Chem..

[76] Klatt P., Molina E.P., Lamas S. (1999). Nitric oxide inhibits c-Jun DNA binding by specifically targeted S-glutathionylation. J. Biol. Chem..

[77] Giustarini D., Milzani A., Aldini G., Carini M., Rossi R., Dalle-Donne I. (2005). S-nitrosation versus S-glutathionylation of protein sulfhydryl groups by S-nitrosoglutathione. Antioxid. Redox Signal..

[78] Mohr S., Hallak H., de Boitte A., Lapetina E.G., Brüne B. (1999). Nitric oxide-induced S-glutathionylation and inactivation of glyceraldehyde-3-phosphate dehydrogenase. J. Biol. Chem..

[79] Dutka T.L., Mollica J.P., Lamboley C.R., Weerakkody V.C., Greening D.W., Posterino G.S., Murphy R.M., Lamb G.D. (2017). *S*-nitrosylation and *S*-glutathionylation of Cys134 on troponin I have opposing competitive actions on Ca^2+^ sensitivity in rat fast-twitch muscle fibers. Am. J. Physiol. Cell Physiol..

[80] Huang K.P., Huang F.L. (2002). Glutathionylation of proteins by glutathione disulfide S-oxide. Biochem. Pharmacol..

[81] Li J., Huang F.L., Huang K.P. (2001). Glutathiolation of proteins by glutathione disulfide S-oxide derived from S-nitrosoglutathione. Modifications of rat brain neurogranin/RC3 and neuromodulin/GAP-43. J. Biol. Chem..

[82] Okamoto T., Akaike T., Sawa T., Miyamoto Y., van der Vliet A., Maeda H. (2001). Activation of matrix metalloproteinases by peroxynitrite-induced protein S-glutathiolation via disulfide S-oxide formation. J. Biol. Chem..

[83] Sadidi M., Geddes T.J., Kuhn D.M. (2005). S-thiolation of tyrosine hydroxylase by reactive nitrogen species in the presence of cysteine or glutathione. Antioxid. Redox Signal..

[84] Boyland E., Chasseaud L.F. (1967). Enzyme-catalysed conjugations of glutathione with unsaturated compounds. Biochem. J..

[85] Townsend D.M., Manevich Y., He L., Hutchens S., Pazoles C.J., Tew K.D. (2009). Novel role for glutathione S-transferase pi. Regulator of protein S-Glutathionylation following oxidative and nitrosative stress. J. Biol. Chem..

[86] Manevich Y., Feinstein S.I., Fisher A.B. (2004). Activation of the antioxidant enzyme 1-CYS peroxiredoxin requires glutathionylation mediated by heterodimerization with pi GST. Proc. Natl. Acad. Sci. USA.

[87] Bartolini D., Torquato P., Piroddi M., Galli F. (2019). Targeting glutathione S-transferase P and its interactome with selenium compounds in cancer therapy. Biochim. Biophys. Acta Gen. Subj..

[88] Townsend D.M., He L., Hutchens S., Garrett T.E., Pazoles C.J., Tew K.D. (2008). NOV-002, a glutathione disulfide mimetic, as a modulator of cellular redox balance. Cancer Res..

[89] Ko K.Y., Lee J.H., Jang J.K., Jin Y., Kang H., Kim I.Y. (2019). S-Glutathionylation of mouse selenoprotein W prevents oxidative stress-induced cell death by blocking the formation of an intramolecular disulfide bond. Free Radic. Biol. Med..

[90] Ye Z.W., Zhang J., Ancrum T., Manevich Y., Townsend D.M., Tew K.D. (2017). Glutathione S-Transferase P-Mediated Protein S-Glutathionylation of Resident Endoplasmic Reticulum Proteins Influences Sensitivity to Drug-Induced Unfolded Protein Response. Antioxid. Redox Signal..

[91] Carvalho A.N., Marques C., Guedes R.C., Castro-Caldas M., Rodrigues E., van Horssen J., Gama M.J. (2016). S-Glutathionylation of Keap1: A new role for glutathione S-transferase pi in neuronal protection. FEBS Lett..

[92] Zhang J., Ye Z.W., Chen W., Manevich Y., Mehrotra S., Ball L., Janssen-Heininger Y., Tew K.D., Townsend D.M. (2018). *S*-Glutathionylation of estrogen receptor α affects dendritic cell function. J. Biol. Chem..

[93] Uemura T., Tsaprailis G., Gerner E.W. (2019). GSTΠ stimulates caveolin-1-regulated polyamine uptake via actin remodeling. Oncotarget.

[94] Klaus A., Zorman S., Berthier A., Polge C., Ramirez S., Michelland S., Sève M., Vertommen D., Rider M., Lentze N. (2013). Glutathione S-transferases interact with AMP-activated protein kinase: Evidence for S-glutathionylation and activation in vitro. PLoS ONE.

[95] Tew K.D., Townsend D.M. (2011). Regulatory functions of glutathione S-transferase P1-1 unrelated to detoxification. Drug Metab. Rev..

[96] Fujitani N., Yoneda A., Takahashi M., Takasawa A., Aoyama T., Miyazaki T. (2019). Silencing of Glutathione S-Transferase Pi Inhibits Cancer Cell Growth via Oxidative Stress Induced by Mitochondria Dysfunction. Sci. Rep..

[97] Janssen-Heininger Y.M., Nolin J.D., Hoffman S.M., van der Velden J.L., Tully J.E., Lahue K.G., Abdalla S.T., Chapman D.G., Reynaert N.L., van der Vliet A. (2013). Emerging mechanisms of glutathione-dependent chemistry in biology and disease. J. Cell Biochem..

[98] Ercolani L., Scirè A., Galeazzi R., Massaccesi L., Cianfruglia L., Amici A., Piva F., Urbanelli L., Emiliani C., Principato G. (2016). A possible S-glutathionylation of specific proteins by glyoxalase II: An in vitro and in silico study. Cell Biochem. Funct..

[99] Galeazzi R., Laudadio E., Falconi E., Massaccesi L., Ercolani L., Mobbili G., Minnelli C., Scirè A., Cianfruglia L., Armeni T. (2018). Protein-protein interactions of human glyoxalase II: Findings of a reliable docking protocol. Org. Biomol. Chem..

[100] Brings S., Fleming T., Freichel M., Muckenthaler M.U., Herzig S., Nawroth P.P. (2017). Dicarbonyls and Advanced Glycation End-Products in the Development of Diabetic Complications and Targets for Intervention. Int. J. Mol. Sci..

[101] Harmel R., Fiedler D. (2018). Features and regulation of non-enzymatic post-translational modifications. Nat. Chem. Biol..

[102] Ren G., Stephan D., Xu Z., Zheng Y., Tang D., Harrison R.S., Kurz M., Jarrott R., Shouldice S.R., Hiniker A. (2009). Properties of the thioredoxin fold superfamily are modulated by a single amino acid residue. J. Biol. Chem..

[103] Zimmermann J., Oestreicher J., Hess S., Herrmann J.M., Deponte M., Morgan B. (2020). One cysteine is enough: A monothiol Grx can functionally replace all cytosolic Trx and dithiol Grx. Redox Biol..

[104] Chrestensen C.A., Starke D.W., Mieyal J.J. (2000). Acute cadmium exposure inactivates thioltransferase (Glutaredoxin), inhibits intracellular reduction of protein-glutathionyl-mixed disulfides, and initiates apoptosis. J. Biol. Chem..

[105] Jung C.H., Thomas J.A. (1996). S-glutathiolated hepatocyte proteins and insulin disulfides as substrates for reduction by glutaredoxin, thioredoxin, protein disulfide isomerase, and glutathione. Arch. Biochem. Biophys..

[106] Herrero E., de la Torre-Ruiz M.A. (2007). Monothiol glutaredoxins: A common domain for multiple functions. Cell. Mol. Life Sci..

[107] Couturier J., Przybyla-Toscano J., Roret T., Didierjean C., Rouhier N. (2015). The roles of glutaredoxins ligating Fe-S clusters: Sensing, transfer or repair functions?. Biochim. Biophys. Acta.

[108] Berndt C., Lillig C.H., Holmgren A. (2008). Thioredoxins and glutaredoxins as facilitators of protein folding. Biochim. Biophys. Acta.

[109] Park J.W., Mieyal J.J., Rhee S.G., Chock P.B. (2009). Deglutathionylation of 2-Cys peroxiredoxin is specifically catalyzed by sulfiredoxin. J. Biol. Chem..

[110] Findlay V.J., Tapiero H., Townsend D.M. (2005). Sulfiredoxin: A potential therapeutic agent?. Biomed. Pharmacother..

[111] Findlay V.J., Townsend D.M., Morris T.E., Fraser J.P., He L., Tew K.D. (2006). A novel role for human sulfiredoxin in the reversal of glutathionylation. Cancer Res..

[112] Shao D., Fry J.L., Han J., Hou X., Pimentel D.R., Matsui R., Cohen R.A., Bachschmid M.M. (2014). A redox-resistant sirtuin-1 mutant protects against hepatic metabolic and oxidant stress. J. Biol. Chem..

[113] Mandato A., Chai Y.C. (2018). Regulation of antigen 85C activity by reversible S-glutathionylation. IUBMB Life.

[114] Gandhirajan R.K., Jain M., Walla B., Johnsen M., Bartram M.P., Huynh Anh M., Rinschen M.M., Benzing T., Schermer B. (2016). Cysteine S-Glutathionylation Promotes Stability and Activation of the Hippo Downstream Effector Transcriptional Co-activator with PDZ-binding Motif (TAZ). J. Biol. Chem..

[115] Nagarkoti S., Dubey M., Sadaf S., Awasthi D., Chandra T., Jagavelu K., Kumar S., Dikshit M. (2019). Catalase S-Glutathionylation by NOX2 and Mitochondrial-Derived ROS Adversely Affects Mice and Human Neutrophil Survival. Inflammation.

[116] Sánchez G., Pedrozo Z., Domenech R.J., Hidalgo C., Donoso P. (2005). Tachycardia increases NADPH oxidase activity and RyR2 S-glutathionylation in ventricular muscle. J. Mol. Cell. Cardiol..

[117] Yang J., Zhang H., Gong W., Liu Z., Wu H., Hu W., Chen X., Wang L., Wu S., Chen C. (2020). *S*-Glutathionylation of human inducible Hsp70 reveals a regulatory mechanism involving the C-terminal α-helical lid. J. Biol. Chem..

[118] Porter C.M., Truman A.W., Truttmann M.C. (2020). Post-translational modifications of Hsp70 family proteins: Expanding the chaperone code. J. Biol. Chem..

[119] Zhang J., Ye Z.W., Chen W., Culpepper J., Jiang H., Ball L.E., Mehrotra S., Blumental-Perry A., Tew K.D., Townsend D.M. (2020). Altered Redox Regulation and S-Glutathionylation of BiP Contribute to Bortezomib Resistance in Multiple Myeloma *Free Radic*. Biol. Med..

[120] Gething M.J. (1999). Role and regulation of the ER chaperone BiP. Semin. Cell Dev. Biol..

[121] Pizzino G., Irrera N., Cucinotta M., Pallio G., Mannino F., Arcoraci V., Squadrito F., Altavilla D., Bitto A. (2017). Oxidative Stress: Harms and Benefits for Human Health. Oxid. Med. Cell. Longev..

[122] Zhou L., Chan J.C.Y., Chupin S., Gueguen N., Desquiret-Dumas V., Koh S.K., Li J., Gao Y., Deng L., Verma C. (2020). Increased Protein *S*-Glutathionylation in Leber’s Hereditary Optic Neuropathy (LHON). Int. J. Mol. Sci..

[123] Jeon D., Park H.J., Kim H.S. (2018). Protein S-glutathionylation induced by hypoxia increases hypoxia-inducible factor-1α in human colon cancer cells. Biochem. Biophys. Res. Commun..

[124] Anathy V., Lahue K.G., Chapman D.G., Chia S.B., Casey D.T., Aboushousha R., van der Velden J., Elko E., Hoffman S.M., McMillan D.H. (2018). Reducing protein oxidation reverses lung fibrosis. Nat. Med..

[125] Tamma G., Valenti G. (2016). Evaluating the Oxidative Stress in Renal Diseases: What Is the Role for S-Glutathionylation?. Antioxid. Redox Signal..

[126] Maki K., Nagai K., Suzuki M., Inomata T., Yoshida T., Nishimura M. (2015). Temporal changes in glutaredoxin 1 and protein s-glutathionylation in allergic airway inflammation. PLoS ONE.

[127] Hong C., Seo H., Kwak M., Jeon J., Jang J., Jeong E.M., Myeong J., Hwang Y.J., Ha K., Kang M.J. (2015). Increased TRPC5 glutathionylation contributes to striatal neuron loss in Huntington’s disease. Brain.

[128] Sánchez-Gómez F.J., Espinosa-Díez C., Dubey M., Dikshit M., Lamas S. (2013). S-glutathionylation: Relevance in diabetes and potential role as a biomarker. Biol. Chem..

[129] Nonaka K., Kume N., Urata Y., Seto S., Kohno T., Honda S., Ikeda S., Muroya T., Ikeda Y., Ihara Y. (2007). Serum levels of S-glutathionylated proteins as a risk-marker for arteriosclerosis obliterans. Circ. J..

[130] Grek C.L., Reyes L., Townsend D.M., Tew K.D. (2014). S-glutathionylation of buccal cell proteins as biomarkers of exposure to hydrogen peroxide. BBA Clin..

[131] Cui H., Kong Y., Zhang H. (2012). Oxidative stress, mitochondrial dysfunction, and aging. J. Signal Transduct..

[132] Harman D. (1956). Aging: A theory based on free radical and radiation chemistry. J. Gerontol..

[133] Zhu Y., Carvey P.M., Ling Z. (2006). Age-related changes in glutathione and glutathione-related enzymes in rat brain. Brain Res..

[134] Cha S.J., Kim H., Choi H.J., Lee S., Kim K. (2017). Protein Glutathionylation in the Pathogenesis of Neurodegenerative Diseases. Oxid. Med. Cell. Longev..

[135] Hodson R. (2018). Alzheimer’s disease. Nature.

[136] Huang W.J., Zhang X., Chen W.W. (2016). Role of oxidative stress in Alzheimer’s disease. Biomed. Rep..

[137] Newman S.F., Sultana R., Perluigi M., Coccia R., Cai J., Pierce W.M., Klein J.B., Turner D.M., Butterfield D.A. (2007). An increase in S-glutathionylated proteins in the Alzheimer’s disease inferior parietal lobule, a proteomics approach. J. Neurosci. Res..

[138] Di Domenico F., Cenini G., Sultana R., Perluigi M., Uberti D., Memo M., Butterfield D.A. (2009). Glutathionylation of the pro-apoptotic protein p53 in Alzheimer’s disease brain: Implications for AD pathogenesis. Neurochem. Res..

[139] Rani P., Krishnan S., Rani Cathrine C. (2017). Study on Analysis of Peripheral Biomarkers for Alzheimer’s Disease Diagnosis. Front. Neurol..

[140] Bonora M., Wieckowski M.R., Sinclair D.A., Kroemer G., Pinton P., Galluzzi L. (2019). Targeting mitochondria for cardiovascular disorders: Therapeutic potential and obstacles. Nat. Rev. Cardiol..

[141] Tahrir F.G., Langford D., Amini S., Mohseni Ahooyi T., Khalili K. (2019). Mitochondrial quality control in cardiac cells: Mechanisms and role in cardiac cell injury and disease. J. Cell. Physiol..

[142] Pastore A., Piemonte F. (2013). Protein glutathionylation in cardiovascular diseases. Int. J. Mol. Sci..

[143] Lu L., Liu M., Sun R., Zheng Y., Zhang P. (2015). Myocardial Infarction: Symptoms and Treatments. Cell Biochem. Biophys..

[144] Eaton P., Wright N., Hearse D.J., Shattock M.J. (2002). Glyceraldehyde phosphate dehydrogenase oxidation during cardiac ischemia and reperfusion. J. Mol. Cell. Cardiol..

[145] Chen F.C., Ogut O. (2006). Decline of contractility during ischemia-reperfusion injury: Actin glutathionylation and its effect on allosteric interaction with tropomyosin. Am. J. Physiol. Cell Physiol..

[146] Chen Y.R., Chen C.L., Pfeiffer D.R., Zweier J.L. (2007). Mitochondrial complex II in the post-ischemic heart: Oxidative injury and the role of protein S-glutathionylation. J. Biol. Chem..

[147] Avner B.S., Shioura K.M., Scruggs S.B., Grachoff M., Geenen D.L., Helseth D.L., Farjah M., Goldspink P.H., Solaro R.J. (2012). Myocardial infarction in mice alters sarcomeric function via post-translational protein modification. Mol. Cell. Biochem..

[148] Alegre-Cebollada J., Kosuri P., Giganti D., Eckels E., Rivas-Pardo J.A., Hamdani N., Warren C.M., Solaro R.J., Linke W.A., Fernández J.M. (2014). S-glutathionylation of cryptic cysteines enhances titin elasticity by blocking protein folding. Cell.

[149] Chakouri N., Reboul C., Boulghobra D., Kleindienst A., Nottin S., Gayrard S., Roubille F., Matecki S., Lacampagne A., Cazorla O. (2018). Stress-induced protein S-glutathionylation and phosphorylation crosstalk in cardiac sarcomeric proteins—Impact on heart function. Int. J. Cardiol..

[150] Murry C.E., Jennings R.B., Reimer K.A. (1986). Preconditioning with ischemia: A delay of lethal cell injury in ischemic myocardium. Circulation.

[151] Domenech R.J., Sánchez G., Donoso P., Parra V., Macho P. (2003). Effect of tachycardia on myocardial sarcoplasmic reticulum and Ca2+ dynamics: A mechanism for preconditioning?. J. Mol. Cell. Cardiol..

[152] Nikolaienko R., Bovo E., Zima A.V. (2018). Redox Dependent Modifications of Ryanodine Receptor: Basic Mechanisms and Implications in Heart Diseases. Front. Physiol..

[153] Sánchez G., Escobar M., Pedrozo Z., Macho P., Domenech R., Härtel S., Hidalgo C., Donoso P. (2008). Exercise and tachycardia increase NADPH oxidase and ryanodine receptor-2 activity: Possible role in cardioprotection. Cardiovasc. Res..

[154] Shimizu I., Minamino T. (2016). Physiological and pathological cardiac hypertrophy. J. Mol. Cell. Cardiol..

[155] Nakamura M., Sadoshima J. (2018). Mechanisms of physiological and pathological cardiac hypertrophy. Nat. Rev. Cardiol..

[156] Bueno O.F., De Windt L.J., Tymitz K.M., Witt S.A., Kimball T.R., Klevitsky R., Hewett T.E., Jones S.P., Lefer D.J., Peng C.F. (2000). The MEK1-ERK1/2 signaling pathway promotes compensated cardiac hypertrophy in transgenic mice. EMBO J..

[157] Gallo S., Vitacolonna A., Bonzano A., Comoglio P., Crepaldi T. (2019). ERK: A Key Player in the Pathophysiology of Cardiac Hypertrophy. Int. J. Mol. Sci..

[158] Pimentel D.R., Adachi T., Ido Y., Heibeck T., Jiang B., Lee Y., Melendez J.A., Cohen R.A., Colucci W.S. (2006). Strain-stimulated hypertrophy in cardiac myocytes is mediated by reactive oxygen species-dependent Ras S-glutathiolation. J. Mol. Cell. Cardiol..

[159] Adachi T., Pimentel D.R., Heibeck T., Hou X., Lee Y.J., Jiang B., Ido Y., Cohen R.A. (2004). S-glutathiolation of Ras mediates redox-sensitive signaling by angiotensin II in vascular smooth muscle cells. J. Biol. Chem..

[160] Panieri E., Santoro M.M. (2016). ROS homeostasis and metabolism: A dangerous liason in cancer cells. Cell Death Dis..

[161] Gorrini C., Harris I.S., Mak T.W. (2013). Modulation of oxidative stress as an anticancer strategy. Nat. Rev. Drug Discov..

[162] Garg R., Benedetti L.G., Abera M.B., Wang H., Abba M., Kazanietz M.G. (2014). Protein kinase C and cancer: What we know and what we do not. Oncogene.

[163] Steinberg S.F. (2015). Mechanisms for redox-regulation of protein kinase C. Front. Pharmacol..

[164] Chu F., Ward N.E., O’Brian C.A. (2003). PKC isozyme S-cysteinylation by cystine stimulates the pro-apoptotic isozyme PKC delta and inactivates the oncogenic isozyme PKC epsilon. Carcinogenesis.

[165] Humphries K.M., Juliano C., Taylor S.S. (2002). Regulation of cAMP-dependent protein kinase activity by glutathionylation. J. Biol. Chem..

[166] Humphries K.M., Deal M.S., Taylor S.S. (2005). Enhanced dephosphorylation of cAMP-dependent protein kinase by oxidation and thiol modification. J. Biol. Chem..

[167] Cruz C.M., Rinna A., Forman H.J., Ventura A.L., Persechini P.M., Ojcius D.M. (2007). ATP activates a reactive oxygen species-dependent oxidative stress response and secretion of proinflammatory cytokines in macrophages. J. Biol. Chem..

[168] Rao R.K., Clayton L.W. (2002). Regulation of protein phosphatase 2A by hydrogen peroxide and glutathionylation. Biochem. Biophys. Res. Commun..

[169] Velu C.S., Niture S.K., Doneanu C.E., Pattabiraman N., Srivenugopal K.S. (2007). Human p53 is inhibited by glutathionylation of cysteines present in the proximal DNA-binding domain during oxidative stress. Biochemistry.

[170] Pineda-Molina E., Klatt P., Vázquez J., Marina A., García de Lacoba M., Pérez-Sala D., Lamas S. (2001). Glutathionylation of the p50 subunit of NF-kappaB: A mechanism for redox-induced inhibition of DNA binding. Biochemistry.

[171] Qanungo S., Starke D.W., Pai H.V., Mieyal J.J., Nieminen A.L. (2007). Glutathione supplementation potentiates hypoxic apoptosis by S-glutathionylation of p65-NFkappaB. J. Biol. Chem..

[172] Butturini E., Darra E., Chiavegato G., Cellini B., Cozzolino F., Monti M., Pucci P., Dell’Orco D., Mariotto S. (2014). S-Glutathionylation at Cys328 and Cys542 impairs STAT3 phosphorylation. ACS Chem. Biol..

[173] Hensley P., Mishra M., Kyprianou N. (2013). Targeting caspases in cancer therapeutics. Biol. Chem..

[174] Zamaraev A.V., Kopeina G.S., Prokhorova E.A., Zhivotovsky B., Lavrik I.N. (2017). Post-translational Modification of Caspases: The Other Side of Apoptosis Regulation. Trends Cell Biol..

[175] Boice A., Bouchier-Hayes L. (2020). Targeting apoptotic caspases in cancer. Biochim. Biophys. Acta Mol. Cell Res..

[176] Huang Z., Pinto J.T., Deng H., Richie J.P. (2008). Inhibition of caspase-3 activity and activation by protein glutathionylation. Biochem. Pharmacol..

[177] Pan S., Berk B.C. (2007). Glutathiolation regulates tumor necrosis factor-alpha-induced caspase-3 cleavage and apoptosis: Key role for glutaredoxin in the death pathway. Circ. Res..

[178] Musaogullari A., Mandato A., Chai Y.-C. (2020). Role of Glutathione Depletion and Reactive Oxygen Species Generation of Caspase-3 Activation: A Study with the Kinase Inhibitor Staurosporine. Front. Physiol..

[179] Byrne C.D., Targher G. (2015). NAFLD: A multisystem disease. J. Hepatol..

[180] Friedman S.L., Neuschwander-Tetri B.A., Rinella M., Sanyal A.J. (2018). Mechanisms of NAFLD development and therapeutic strategies. Nat. Med..

[181] Videla L.A., Rodrigo R., Araya J., Poniachik J. (2006). Insulin resistance and oxidative stress interdependency in non-alcoholic fatty liver disease. Trends Mol. Med..

[182] Dou X., Li S., Hu L., Ding L., Ma Y., Ma W., Chai H., Song Z. (2018). Glutathione disulfide sensitizes hepatocytes to TNFα-mediated cytotoxicity via IKK-β S-glutathionylation: A potential mechanism underlying non-alcoholic fatty liver disease. Exp. Mol. Med..

[183] Rufini A., Tucci P., Celardo I., Melino G. (2013). Senescence and aging: The critical roles of p53. Oncogene.

[184] Yu J., Zhang L. (2008). PUMA, a potent killer with or without p53. Oncogene.

[185] Deng X.Q., Chen L.L., Li N.X. (2007). The expression of SIRT1 in nonalcoholic fatty liver disease induced by high-fat diet in rats. Liver Int..

[186] Yin H., Hu M., Liang X., Ajmo J.M., Li X., Bataller R., You M. (2014). Deletion of SIRT1 from hepatocytes in mice disrupts lipin-1 signaling and aggravates alcoholic fatty liver. Gastroenterology.

[187] Seo Y.Y., Cho Y.K., Bae J.C., Seo M.H., Park S.E., Rhee E.J., Park C.Y., Oh K.W., Park S.W., Lee W.Y. (2013). Tumor Necrosis Factor-α as a Predictor for the Development of Nonalcoholic Fatty Liver Disease: A 4-Year Follow-Up Study. Endocrinol. Metab..

[188] Kakino S., Ohki T., Nakayama H., Yuan X., Otabe S., Hashinaga T., Wada N., Kurita Y., Tanaka K., Hara K. (2018). Pivotal Role of TNF-α in the Development and Progression of Nonalcoholic Fatty Liver Disease in a Murine Model. Horm. Metab. Res..

[189] Hayden M.S., Ghosh S. (2014). Regulation of NF-κB by TNF family cytokines. Semin. Immunol..

[190] Solt L.A., May M.J. (2008). The IkappaB kinase complex: Master regulator of NF-kappaB signaling. Immunol. Res..

